# Escores de Risco Poligênico: O Próximo Passo para Melhorar a Estratificação de Risco na Doença Arterial Coronariana?

**DOI:** 10.36660/abc.20240252

**Published:** 2024-09-12

**Authors:** Ricardo Stein, Filipe Ferrari, Diego García-Giustiniani

**Affiliations:** 1 Programa de Pós-Graduação em Cardiologia e Ciências Cardiovasculares Universidade Federal do Rio Grande do Sul Porto Alegre RS Brasil Programa de Pós-Graduação em Cardiologia e Ciências Cardiovasculares, Universidade Federal do Rio Grande do Sul, Porto Alegre, RS – Brasil; 2 Departamento de Medicina Interna Universidade Federal do Rio Grande do Sul Porto Alegre RS Brasil Departamento de Medicina Interna, Universidade Federal do Rio Grande do Sul, Porto Alegre, RS – Brasil; 3 Instituto de Investigación Biomédica de A Coruña Coruña Galicia Espanha Instituto de Investigación Biomédica de A Coruña (INIBIC), Coruña, Galicia – Espanha

**Keywords:** Doença Arterial Coronariana, Risco, Genoma Humano, Doenças Cardiovasculares

## Abstract

Apesar dos avanços significativos no tratamento da doença arterial coronariana (DAC) e das reduções nas taxas de mortalidade anuais nas últimas décadas, a DAC continua sendo a principal causa de morte no mundo. Consequentemente, há uma necessidade contínua de esforços para abordar essa situação. Os algoritmos clínicos atuais para identificar pacientes em risco são particularmente imprecisos para indivíduos de risco moderado. Por esse motivo, foi sugerido que são necessários testes auxiliares, incluindo triagem genética preditiva. À medida que os estudos genéticos se expandem rapidamente e os dados genômicos se tornam mais acessíveis, diversos escores de risco genético têm sido propostos para identificar e avaliar a suscetibilidade de um indivíduo ao desenvolvimento de doenças, incluindo a DAC. De fato, o campo da genética tem contribuído substancialmente para a previsão de risco, particularmente nos casos em que as crianças têm genitores com DAC prematura, resultando em um risco aumentado de até 75%. Os escores de risco poligênico (PRSs, do inglês *polygenic risk scores*) surgiram como uma ferramenta potencialmente valiosa para compreender e estratificar o risco genético de um indivíduo. O PRS é calculado como uma soma ponderada de variantes de nucleotídeo único presentes em todo o genoma humano, identificáveis por meio de estudos de associação genômica ampla, e associadas a várias doenças cardiometabólicas. O uso dos PRSs é promissor, pois permite o desenvolvimento de estratégias personalizadas para prevenir ou diagnosticar patologias específicas de forma precoce. Ademais, seu uso é capaz de complementar os escores clínicos existentes, aumentando a precisão da previsão de risco individual. Consequentemente, a aplicação dos PRSs tem o potencial de impactar positivamente os custos e os desfechos adversos associados à DAC. A presente revisão narrativa oferece uma visão ampla do papel dos PRSs no contexto da DAC.

## Graphical abstract

**Figura Central:**
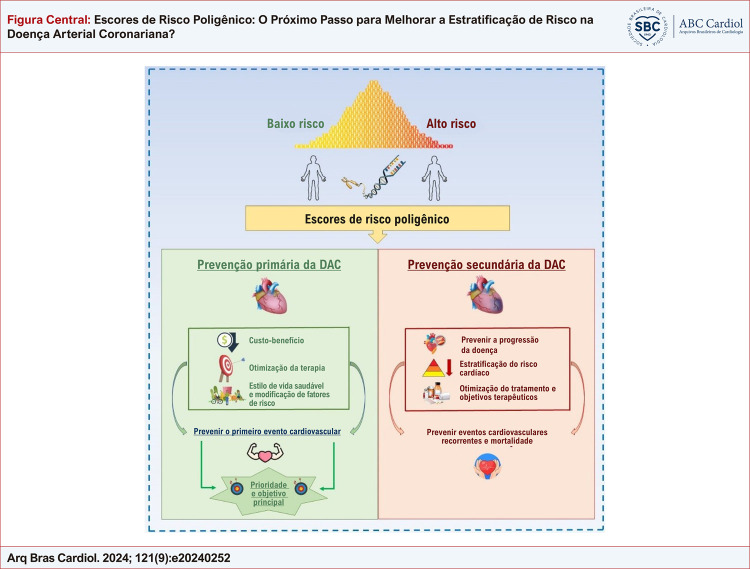
: Escores de Risco Poligênico: O Próximo Passo para Melhorar a Estratificação de Risco na Doença Arterial Coronariana?

Papel dos escores de risco poligênico na prevenção primária e secundária da doença arterial coronariana. DAC: doença arterial coronariana.

## Introdução

Por décadas, vários algoritmos clínicos foram desenvolvidos para identificar pacientes em risco de doença arterial coronariana (DAC) e formular estratégias de prevenção primária. No entanto, esses algoritmos provaram ser menos eficazes em indivíduos com risco intermediário.^1^ Portanto, nos últimos anos, testes adicionais conhecidos como intensificadores de risco ou modificadores de risco têm sido investigados para identificar esse grupo de risco específico com mais precisão. Alguns desses testes auxiliares incluem apolipoproteína B, lipoproteína (a) ou medidas de inflamação, como proteína C-reativa de alta sensibilidade ou escore de cálcio coronariano.^2-6^ Essas ferramentas clínicas devem atender à condição de estarem independentemente associadas à DAC e conferirem pelo menos um risco duas vezes maior de doença.^1,7^ Além das ferramentas clínicas investigadas, resultados anteriores da coorte de descendentes do Framingham Heart Study demonstraram que um histórico de DAC prematura estava associado a um aumento de duas vezes na probabilidade de doença cardiovascular após o ajuste para fatores de risco clínicos tradicionais. Isso sugere uma base hereditária clara para doenças cardiovasculares.^8^

Avanços recentes na identificação de dados genômicos, particularmente nas últimas décadas, apresentaram oportunidades significativas para o desenvolvimento de preditores genéticos independentes. A elucidação da base genética de doenças, apoiada por estudos recentes que revelaram um aumento de quase duas vezes nos níveis de risco entre populações com alto percentil de escore de risco, é promissora para a integração desses escores na prática clínica. Essa integração pode ser alcançada pela incorporação de escores de risco poligênico (PRSs, do inglês *polygenic risk scores*) nos modelos de avaliação de risco existentes.^1^

A presente revisão narrativa aborda a compreensão atual dos PRSs em relação à DAC. Visamos destacar os benefícios e limitações potenciais dos PRSs para avaliação de risco de DAC, ao mesmo tempo em que consideramos a sua custo-efetividade.

## Métodos

Empregamos uma abordagem narrativa, permitindo uma síntese qualitativa de estudos relevantes. As informações extraídas foram então organizadas para construir uma narrativa coesa sobre o papel de PRSs no contexto do diagnóstico e manejo de DAC. Uma busca bibliográfica no PubMed/MEDLINE foi realizada para estudos publicados desde o início até 26 de março de 2024. Foi utilizada a seguinte estratégia de busca em inglês: (polygenic risk scores OU genetic risk scores) e (coronary artery disease OU CAD). Realizamos uma busca manual nas listas de referências de todos os estudos incluídos para identificar outros artigos potenciais. Dos 1.017 artigos identificados, 19 foram incluídos, compreendendo 15 estudos de coorte, 3 análises de custo-efetividade e 1 diretriz.

### Escores de risco poligênico – definição e conceito

O PRS é uma soma calculada de alelos de risco portados por um indivíduo, servindo como uma ferramenta preditiva para avaliar o risco.^9^ Resumidamente, PRSs são medidas que quantificam a predisposição genética de um indivíduo a uma doença, considerando múltiplas variantes genéticas em todo o genoma. Esses escores integram informações de um grande número de marcadores genéticos, cada um contribuindo com uma pequena porção para o risco total. Sua construção envolve o uso e a validação de variantes de nucleotídeo único (SNVs, do inglês *single-nucleotide variants*), representando alterações de um único nucleotídeo em uma posição específica no genoma, obtidas principalmente a partir de estudos de associação genômica ampla (GWAS, do inglês *genome-wide association studies*).

Em termos mais simples, um PRS é um algoritmo computacional que incorpora informações derivadas de SNVs. Inicialmente, os escores de risco eram baseados em cálculos não ponderados, mas essa abordagem foi substituída por escores ponderados, reconhecendo os efeitos variados de SNVs individuais em doenças específicas.^10^ Os PRSs por si só não levam em conta fatores críticos envolvidos no desenvolvimento de uma doença específica, por exemplo, influências ambientais. Portanto, deve ser considerado como um dos vários riscos independentes contribuintes e não pode prever definitivamente se um indivíduo desenvolverá a doença.^11^

Para fornecer contexto, é essencial entender que, mesmo que um indivíduo possua uma forte predisposição genética para o vício em drogas, isso se torna irrelevante se ele nunca iniciar o uso de drogas.^12^

### Construção de Escores de Risco Poligênico

Essencialmente, um PRS é construído com base em GWAS, usando polimorfismos de nucleotídeo único (SNPs, do inglês *single-nucleotide polymorphisms*) genotipados. Cada SNP tem atributos, incluindo identificador, posição, classificação de risco, tamanho do efeito, medida de confiança e valor p. O cálculo do PRS envolve a soma de alelos de risco (0, 1 ou 2) de dados alvo, ponderados pelos tamanhos de efeito dos alelos de risco. Os tamanhos de efeito são representados como log (*odds ratio*) para características binárias ou como declives de regressão para características contínuas.^13^ Após identificar os SNPs e seus pesos, o PRS é calculado somando suas contribuições. Um PRS mais alto indica uma predisposição genética mais alta, enquanto pontuações mais baixas sugerem um risco menor.

PRSs ponderados são preferíveis devido à melhor precisão preditiva em relação aos PRSs não ponderados.^14^ O limiar de valor p escolhido determina o número de SNPs em um modelo de PRS, impactando a sensibilidade e a especificidade na predição. Portanto, é comum testar diferentes tamanhos de PRS com vários limites de valor p durante o desenvolvimento.

Outra consideração crucial é avaliar se as variantes no modelo estão em desequilíbrio de ligação (LD, do inglês *linkage disequilibrium*), indicando co-herança. O ajuste de LD ajuda a evitar a super-representação de variantes genéticas em regiões de alto LD, sendo essencial para o desempenho dos PRSs. Ferramentas computacionais modernas como LDPred e meta-genetic risk score (metaGRS) abordam isso.^15,16^ Por exemplo, metaGRS oferece melhor discriminação de risco em comparação com calculadoras mais antigas baseadas em SNPs selecionados, capturando uma proporção maior de herdabilidade de DAC, em torno de 27%, com melhorias significativas na previsão de risco.^15^

**Figura 1:**
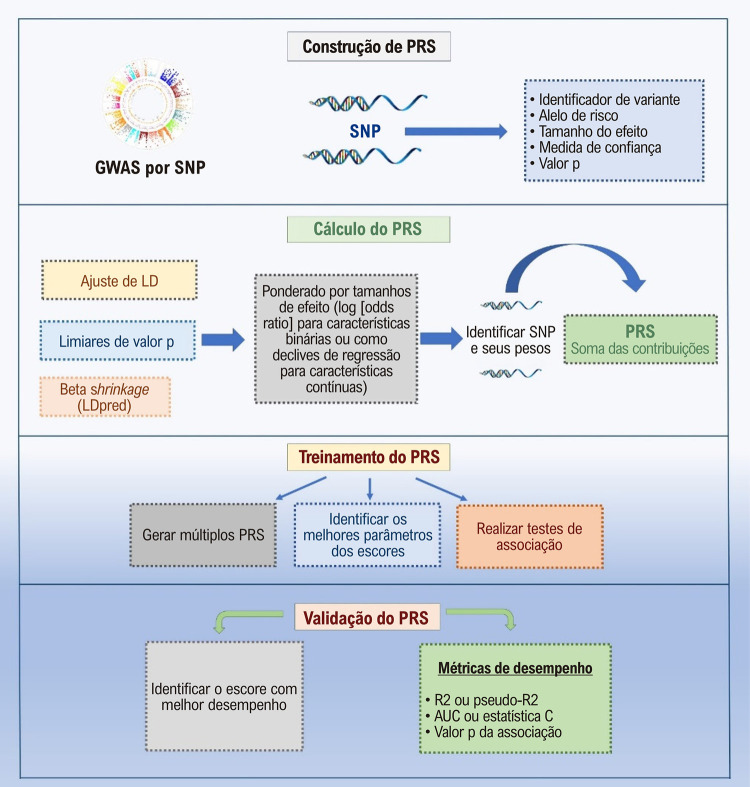
– Aspectos essenciais de uma análise de escore de risco poligênico. AUC: área sob a curva ROC (receiver operator characteristic); GWAS: estudos de associação genômica ampla; LD: desequilíbrio de ligação; PRS: escore de risco poligênico; SNP: polimorfismos de nucleotídeo único.

O próximo passo trata de identificar o modelo de PRS ideal testando vários limiares de valor p e avaliando diferentes modelos por meio de estudos de associação. O escore com melhor desempenho é então validado usando medidas epidemiológicas padrão, como *odds ratios* ou *hazards ratios* por desvio padrão (DP) de mudança no PRS (para doenças binárias), a proporção de variação fenotípica explicada (R2 ou pseudo-R2), a área sob a curva ROC (*receiver operator characteristic*) ou estatística C e o valor p de associação.^17^ Essa abordagem permite a classificação de indivíduos nas categorias de baixo, intermediário ou alto risco. Indivíduos com menos polimorfismos relacionados à doença geralmente se enquadram na categoria de baixo risco. É importante observar que os PRSs podem variar entre diferentes doenças e populações. A Figura 1 resume os aspectos essenciais de uma análise de PRS.

### Predisposição genética para doença arterial coronariana

Fatores genéticos contribuem significativamente para o desenvolvimento de DAC, com evidências que datam da década de 1950, quando destacaram a importância de fatores hereditários a esse respeito.^18^ Nesse contexto, cerca de um terço dos pacientes com DAC apresentam histórico familiar positivo, correlacionando-se com um risco aproximadamente 1,5 vez maior de DAC ao longo de suas vidas.^19,20^Um estudo em larga escala do Framingham Heart Study envolvendo mais de 2.300 homens e mulheres com idade média de 44 anos mostrou um risco significativamente maior de eventos cardiovasculares para indivíduos com um genitor que teve doença cardiovascular de início precoce (pai antes dos 55 anos, mãe antes dos 65 anos).^8^ Esse achado se alinha ao conceito de que um histórico familiar de doença cardiovascular de início precoce (antes dos 50 anos) está associado a um risco maior de morte por DAC. Um estudo recente de Taylor et al. oferece corroboração adicional, com mais de 6.200 participantes acompanhados por uma média de 15 anos.^21^ Em seu estudo, mais de 40% dos participantes tinham pelo menos um dos genitores com histórico de doença cardiovascular, enquanto pouco mais da metade não tinha histórico familiar conhecido. Os resultados demonstraram que o histórico familiar de doença cardiovascular aumentou o risco de desenvolver doença cardiovascular futura em 1,7 vez (*hazard ratio* [HR]: 1,71; intervalo de confiança [IC] de 95%, 1,33 a 2,21; p < 0,001).

Ademais, a relação entre o histórico parental de DAC e o risco de desenvolver a doença foi coletada pela associação observada entre DAC e outros fatores de risco comuns, como pressão alta, níveis elevados de colesterol e tabagismo. Também foram encontradas evidências que sugerem uma correlação entre histórico familiar de DAC e marcadores de aterosclerose subclínica (por exemplo, cálcio da artéria coronária, espessura íntima da carótida e função vascular), mesmo após a consideração de fatores de risco tradicionais.^22^ Isso sugere que esses indivíduos podem ser identificados como fortes candidatos para avaliação de doença cardiovascular subclínica, auxiliando assim na melhoria das metas de tratamento e manejo de risco.

Esses dados sugerem que o histórico familiar de DAC é significativo como um fator de risco que deve ser avaliado em conjunto com outros fatores de risco reconhecidos ao determinar a probabilidade de desenvolver DAC.

### Escores de Risco Poligênico como ferramenta complementar na prevenção primária da doença arterial coronariana

#### Identificação de indivíduos em risco de doença arterial coronariana

A identificação de indivíduos em prevenção primária que apresentam alto risco de DAC é crucial, pois permite melhor triagem ou terapias preventivas. O reconhecimento do risco cardiovascular representa o primeiro passo para determinar a abordagem do tratamento individual para prevenção primária. Nesse contexto, várias ferramentas foram criadas para auxiliar nessa avaliação, incluindo PRSs.

Tikkanen et al.^23^ desenvolveram um escore de risco genético e avaliaram sua correlação com eventos de doenças cardiovasculares incidentes ao longo de 12 anos. Analisando 28 variantes genéticas associadas à DAC em uma amostra de mais de 24.000 indivíduos de 4 coortes prospectivas populacionais, os autores compararam o escore de risco genético com fatores de risco convencionais e histórico familiar. O estudo revelou que o escore de risco genético aumentou significativamente a capacidade de prever o risco de DAC para novos eventos. Indivíduos entre os 10% mais altos no escore de risco genético, com base em 28 SNPs apresentaram um aumento de duas vezes no risco de DAC em comparação com aqueles entre os 20% do meio.

No estudo de Khera et al.,^24^ foi calculado um conjunto de PRS em todo o genoma usando o algoritmo LDPred para várias doenças, incluindo DAC. O 1% mais alto na distribuição das 6,6 milhões de variantes de PRS teve quase cinco vezes mais chances de desenvolver DAC (*odds ratio*: 4,83; IC de 95%, 4,25 a 5,46). A *odds ratio* foi calculada comparando aqueles com escores poligênicos elevados em todo o genoma (um preditor quantitativo de risco herdado) com o resto da população em um modelo de regressão logística ajustado para idade, sexo, matriz de genotipagem e os quatro primeiros componentes principais da ancestralidade. Inouye et al.^25^ realizaram uma metanálise de aproximadamente 500.000 indivíduos para desenvolver um escore de risco genômico para DAC com base em mais de 1,7 milhão de variantes genéticas. Os autores verificaram que essas variantes eram responsáveis por 26,8% da herdabilidade da doença. Ademais, seus resultados revelaram que indivíduos classificados no quintil superior do PRS (entre os 20% mais altos) apresentaram um risco quatro vezes maior de DAC (HR: 4,17) em comparação com aqueles entre os 20% mais baixos. O estudo também demonstrou que o escore de risco genômico combinado provou ser um preditor mais confiável de DAC em comparação com vários fatores de risco bem estabelecidos, incluindo colesterol alto e hipertensão. Tanto o escore poligênico de todo o genoma quanto o escore metaGRS foram validados na população franco-canadense.^15^ Os autores confirmaram que os PRSs foram capazes de identificar aproximadamente 6% a 7% da população com risco igual ou maior de DAC do que portadores de uma mutação monogênica de hipercolesterolemia familiar.^15^ Esses resultados significativos ressaltam as capacidades preditivas complementares do PRS juntamente com os fatores de risco estabelecidos para DAC, em vez de promover sua substituição.

Lu et al.^26^ desenvolveram um PRS com 540 variantes genéticas para DAC, avaliando sua utilidade clínica na prevenção primária em um conjunto de treinamento com 2.800 casos de DAC e 2.055 controles. Durante um acompanhamento médio de 13 anos, 1.303 casos incidentes de DAC foram identificados. Aqueles com PRS elevado (entre os 20% mais altos) tiveram um risco aproximadamente 3 vezes maior de DAC em comparação com aqueles entre os 20% mais baixos (HR: 2,91; IC de 95%, 2,43 a 3,49). Adicionar o PRS ao escore de risco clínico resultou em uma melhora modesta, mas significativa, na estatística C (1%) e uma melhora ajustada na reclassificação (3,5%).^26^ Esse estudo destacou o potencial considerável de identificar indivíduos de alto risco para intervenções direcionadas na prática clínica.

Um PRS extremamente elevado para DAC também pode desempenhar um papel importante na terapia precoce com estatina. Mega et al.^27^ investigaram a associação entre um escore de risco baseado em 27 variantes genéticas e a ocorrência de eventos de DAC novos ou recorrentes, considerando preditores clínicos. Os pesquisadores coletaram dados de uma coorte baseada na comunidade e quatro grandes ensaios clínicos randomizados (JUPITER, ASCOT, CARE e PROVE IT-TIME 22), envolvendo mais de 48.000 indivíduos e quase 3.500 eventos.^27^ Entre os indivíduos nas populações de prevenção primária, riscos mais altos de incidência de DAC foram observados nas categorias de risco genético intermediário e alto, com taxas de risco de 1,31 e 1,72, respectivamente, em comparação com a categoria de baixo risco genético (p < 0,0001 para ambos). É interessante notar que o estudo também verificou que a terapia com estatina reduziu significativamente o risco relativo de eventos na categoria de alto risco genético em comparação com a categoria de baixo risco (48% versus 13%, respectivamente). Além disso, um aumento de três vezes no número necessário para tratar (NNT) foi observado nos estudos de prevenção primária entre indivíduos nas categorias de risco genético mais alto e aqueles na categoria de risco genético mais baixo. Especificamente, entre os participantes do ensaio JUPITER inscritos na prevenção primária, o NNT para prevenir um evento cardiovascular isquêmico ao longo de 10 anos foi de 66, 42 e 25 para indivíduos nos grupos de pontuação de risco genético baixo, intermediário e alto, respectivamente. Esses achados destacam que indivíduos com maior risco genético para DAC recebem os benefícios clínicos mais significativos do uso de estatinas. Além disso, o estudo de Tada et al.^28^ indica uma vantagem potencialmente maior na utilização de PRS para indivíduos jovens. Nessa pesquisa, a incorporação de 23 SNPs em um PRS aumentou a previsão do risco de doença cardíaca isquêmica com um acompanhamento mediano de 14,4 anos, independentemente do histórico familiar de doença cardiovascular autorrelatada. O estudo envolveu 23.595 participantes do Malmö Diet and Cancer Study, um estudo prospectivo de base populacional.

Outro estudo baseado em achados sobre as estatinas e a DAC demonstrou que um PRS para DAC tinha uma capacidade preditiva mais forte em indivíduos mais jovens.^29^ O PRS para DAC consistia em 241 variações genéticas significativas distribuídas pelo genoma. Para estimar o risco de doença cardiovascular aterosclerótica em 10 anos, os pesquisadores empregaram equações de coorte agrupadas, que classificaram os indivíduos em categorias de risco baixo (< 5%), limítrofe (5 a < 7,5%), intermediário (7,5 a < 20%) ou alto (≥ 20%). A análise revelou uma forte associação entre o PRS para DAC e o risco de infarto do miocárdio em todas as faixas etárias. Entretanto, o poder preditivo foi notavelmente mais robusto em indivíduos mais jovens (idade < 50 anos: HR por 1 DP de PRS, 1,72; IC de 95%, 1,56 a 1,89; idade de 50 a 60 anos: HR, 1,46; IC de 95%, 1,38 a 1,53; idade > 60 anos: HR, 1,42; IC de 95%, 1,37 a 1,48; p para interação < 0,001). Em pacientes com menos de 50 anos, aqueles com PRS alto apresentaram um risco três a quatro vezes maior de infarto do miocárdio em comparação com aqueles na categoria de PRS baixo.^29^ Consistente com esses achados, um estudo que incluiu participantes do ensaio clínico randomizado controlado de prevenção primária com terapia com estatina (WOSCOPS; N = 4.910) e dois estudos de coorte observacionais (CARDIA e BioImage; N = 1.154 e N = 4.392, respectivamente)^30^ relatou que indivíduos com alto risco genético tinham uma carga maior de aterosclerose subclínica e obtiveram maiores benefícios relativos e absolutos da terapia com estatina para prevenir um primeiro evento de DAC. Além disso, a Diretriz Americana para Prevenção Primária de DAC recomenda terapia preventiva com estatina para indivíduos portadores de uma mutação rara de hipercolesterolemia familiar monogênica.^7^

Resultados de um estudo envolvendo uma coorte do mundo real em prevenção primária apoiam a racionalização do uso de PRS para CAD como uma ferramenta de medicina de precisão para otimizar ainda mais o equilíbrio risco-benefício da terapia com estatina em combinação com fatores de risco tradicionais para doença cardiovascular. A magnitude da eficácia da estatina tornou-se progressivamente mais robusta em grupos com PRS baixo (quintil 1; HR: 0,67; IC de 95%, 0,47 a 0,97), intermediário (quintis 2 a 4; HR: 0,56; IC de 95%, 0,47 a 0,66) e alto (quintil 5; HR: 0,41; IC de 95%, 0,31 a 0,53), com um benefício menor das estatinas no grupo com PRS baixo (p = 0,01 comparando alto versus baixo).^31^

Finalmente, um estudo de autópsia investigou as conexões entre o PRS e a gravidade da aterosclerose em um nível histopatológico em indivíduos que sofreram morte súbita. Ao analisar uma amostra composta por mais de 900 casos, com idade média de 48 anos, os autores verificaram que aqueles no quintil mais alto do PRS tinham aterosclerose mais grave em comparação com aqueles no quintil mais baixo, bem como maior incidência de calcificação e fibroateroma de capa fina, todos achados com significância estatística.^32^ Mesmo após o ajuste para fatores de risco tradicionais de DAC, aqueles no quintil mais alto do PRS tinham maiores chances de ter aterosclerose grave (definida como estenose ≥ 75%; *odds ratio* ajustada: 3,8; IC de 95%, 2,1 a 6,8; p < 0,001) e ruptura de placa (*odds ratio* ajustada: 4,1; IC de 95%, 2,3 a 7,2; p < 0,001). Ademais, indivíduos no quintil mais alto tinham maior probabilidade de ter causas de morte associadas à DAC, especialmente entre pessoas mais jovens (idade ≤ 50 anos; *odds ratio* ajustada: 4,1; IC de 95%, 2,0 a 8,3; p < 0,001).^32^ Esses resultados fornecem evidências sólidas de uma associação entre PRS e aterosclerose avançada, sugerindo que o PRS pode servir como uma ferramenta valiosa para estratificar o risco de DAC, especialmente em populações mais jovens.

## Escores de Risco Poligênico na prevenção secundária de doença arterial coronariana

A prevenção secundária de doença cardíaca coronária visa prevenir a recorrência de eventos coronários. Um alto grau de adesão a medidas de prevenção secundária, especialmente mudanças intensivas no estilo de vida e uso de medicamentos, pode resultar em uma redução significativa na incidência de eventos coronários recorrentes. O PRS, por sua vez, também pode fornecer uma estratégia valiosa para auxiliar na identificação do risco de um novo evento cardiovascular.

Uma coorte do UK Biobank, compreendendo mais de 7.000 adultos de meia-idade (idade média: 62 anos) diagnosticados com DAC estabelecida, foi monitorada por uma duração mediana de aproximadamente 12 anos. Essa investigação identificou o PRS de DAC (índice C: 0,58; IC de 95%, 0,57 a 0,59) como um dos preditores mais robustos de recorrência de DAC. Tanto o histórico de um evento de DAC prematuro inicial quanto um elevado PRS para DAC foram fatores de risco significativos e mutuamente reforçadores para DAC recorrente. É importante notar que o PRS para DAC mostrou uma associação independente com um risco 12% maior de eventos de DAC recorrentes (HR: 1,12; IC de 95%, 1,05 a 1,19).^33^ Conforme destacado em um editorial, é importante ressaltar certas limitações inerentes a esse estudo. Em primeiro lugar, os modelos empregados não tinham uma avaliação da função ventricular esquerda, da presença de insuficiência cardíaca ou doença valvar concomitante, da extensão anatômica da DAC e da carga de doença aterosclerótica associada em outros leitos vasculares. A incorporação dessas variáveis seria capaz de melhorar significativamente a previsão de risco. Ademais, embora a inclusão do PRS tenha contribuído para um índice C geral modesto de 0,676, a capacidade discriminatória exibiu apenas uma melhora limitada e incremental em comparação aos fatores de risco tradicionais sozinhos (índice C de 0,644).^34^ Mesmo com as limitações descritas, esses achados enfatizam o potencial do escore de risco genético tanto na previsão quanto na prevenção de DAC futura.

Em uma coorte de 1.776 pacientes chineses com DAC que foram acompanhados por até 11 anos, a suscetibilidade genética à DAC e seus fatores de risco tradicionais (por exemplo, insuficiência cardíaca, angina, diabetes e colesterol LDL) foram avaliados para prever a morte por todas as causas. Os resultados mostraram que a integração do metaPRS para DAC e seus fatores de risco foi significativamente associada à mortalidade. Nesse estudo, os participantes foram divididos em três grupos com base nos quartis dos escores do metaPRS. Pacientes com DAC no terceiro quartil apresentaram uma incidência cumulativa significativamente maior de mortalidade por todas as causas em comparação com aqueles no primeiro quartil (HR: 3,99; IC de 95%, 2,4 a 6,6) por aumento de DP (p = 9,10 × 10^8^). Além disso, pacientes com DAC e valores intermediários de metaPRS também apresentaram uma incidência cumulativa significativamente maior de mortalidade por todas as causas em comparação com aqueles no primeiro quartil (HR: 2,18; IC de 95%, 1,3 a 3,6) por aumento de DP (p = 2,10 × 10^3^).^35^ Por sua vez, Howe et al.^36^ realizaram um estudo para investigar se o PRS seria capaz de estratificar o risco de eventos subsequentes em sobreviventes de um evento cardíaco isquêmico. Os pesquisadores analisaram duas subamostras do UK Biobank: indivíduos com casos prevalentes de DAC (N = 10.287) e indivíduos sem DAC (N = 393.108) como linha de base. O estudo revelou uma diferença significativa nas associações entre o PRS para DAC e eventos cardiovasculares para indivíduos com e sem DAC anterior. No grupo sem DAC, houve forte evidência de uma associação positiva entre o PRS para DAC e um aumento de 33% no risco de infarto do miocárdio, enquanto o aumento foi de 15% no grupo com DAC. Consequentemente, a utilidade do PRS parece diminuir em indivíduos submetidos à prevenção secundária. Outros autores, incluindo Thompson et al.^37^ também encontraram sucesso limitado na previsão de eventos cardiovasculares futuros (ao longo de um período de acompanhamento de 10 anos) usando escores de risco genético em pacientes com histórico de síndrome coronariana aguda. Esses autores utilizaram SNPs específicos associados à doença cardíaca isquêmica prevalente ou incidente em GWAS, bem como escores de risco genético de 27 SNPs validados com base nessas variantes. As razões subjacentes para essas discrepâncias continuam sem esclarecimento, e pesquisas adicionais são necessárias para determinar a verdadeira contribuição dos escores de risco genético na previsão de risco futuro entre indivíduos que já sofreram um evento cardiovascular.

## Custo-efetividade

Como a aplicação dos PRSs pode aumentar consideravelmente em todo o mundo nas próximas décadas, um tema importante a ser abordado é a custo-efetividade da sua utilização. Portanto, é vital que a aplicação do PRS, além de ser eficaz, seja custo-efetiva o suficiente para permitir seu uso em grande escala.

Kiflen et al.^38^investigaram a relação custo-efetividade do PRS em relação à terapia com estatina, utilizando uma coorte do UK Biobank (N = 96.111; descendência branca e britânica) com risco intermediário de doença cardiovascular, acompanhada durante pelo menos 10 anos. A análise do caso-base, com um custo de genotipagem de US$ 70, resultou em uma relação custo-efetividade incremental de US$ 172.906 por ano de vida ajustado pela qualidade. Na análise de sensibilidade probabilística, a intervenção apresentou uma probabilidade de aproximadamente 50% de ser custo-efetiva a US$ 179.100 por ano de vida ajustado pela qualidade. Esse estudo sugere que incorporar o PRS juntamente com as diretrizes existentes pode ser custo-efetiva para doenças cardiovasculares. Maior previsibilidade combinada com um custo reduzido do PRS poderia melhorar ainda mais a relação custo-efetividade, fornecendo uma base econômica para sua inclusão no atendimento clínico.

Outro estudo envolvendo indivíduos de 40 a 75 anos nos Estados Unidos revelou que a incorporação de um PRS para CAD em um programa de prevenção de doenças cardiovasculares no local de trabalho provou ser custo-efetiva.^39^ A análise mostrou que a inclusão do PRS para CAD incorreu em custos adicionais de mais de US$ 53 por funcionário examinado em comparação ao programa de prevenção cardiovascular no local de trabalho sem o PRS para CAD, e mais de US$ 575 em comparação à ausência de um programa de saúde no local de trabalho. Os autores consequentemente afirmam que a integração de testes poligênicos em um programa de prevenção cardiovascular no local de trabalho não apenas melhora a qualidade de vida dos funcionários, mas também diminui simultaneamente os custos de saúde e atenua as perdas financeiras de produtividade para os empregadores. Os mesmos pesquisadores verificaram que considerar um escore de risco genético (CAD-PRS) para doenças cardíacas levou a melhores resultados em comparação ao uso isolado de uma ferramenta de avaliação de risco padrão. Os pesquisadores observaram uma redução nos custos de saúde por pessoa, melhor qualidade de vida e menos casos de DAC e acidente vascular cerebral.^40^

## Limitações e áreas de incerteza

Apesar de representar uma área promissora e em rápida expansão, os PRSs não estão isentos de limitações, particularmente no que diz respeito à sua validade externa devido ao foco predominante em indivíduos de ascendência europeia. A heterogeneidade entre diferentes populações em todo o mundo dificulta a generalização dos PRSs e sua utilidade em diversos grupos de ancestralidade, o que pode sub-representar certas populações, diminuindo assim a utilidade dos PRSs e limitando a aplicabilidade das ferramentas de previsão de risco.

Um desafio fundamental é a variação potencial na frequência e nos efeitos das variantes genéticas em diferentes populações. Isso é apoiado por achados de pesquisas anteriores.^41^ Além disso, populações com alto grau de mistura, ou mistura de origens ancestrais, tendem a ter maior diversidade genética. Essa complexidade pode dificultar ainda mais a precisão dos PRSs. Para abordar essas questões e melhorar a precisão, é essencial aumentar a diversidade de participantes na pesquisa genômica e desenvolver modelos que levem em conta as variações genéticas específicas dentro de cada grupo populacional.

Consequentemente, conduzir estudos adicionais aplicando PRSs em populações não europeias torna-se imperativo para confirmar ou refutar o papel como um preditor de risco cardiovascular futuro nesses grupos. Uma revisão cienciométrica por Mills e Rahal revelou uma tendência preocupante: embora a diversidade ancestral entre os participantes do GWAS tenha aumentado ao longo do tempo, disparidades significativas permanecem.^42^ Mesmo em 2017, impressionantes 88% dos participantes eram de ascendência europeia. Além disso, um foco geográfico estreito persiste, com 72% das descobertas baseadas em estudos conduzidos em apenas três países: Estados Unidos, Reino Unido e Islândia.^42^ Essas descobertas levantam preocupações sobre um potencial “ciclo de desvantagem” para populações sub-representadas. Apesar dos esforços contínuos, Mills e Rahal enfatizam o desafio contínuo de aumentar a diversidade na pesquisa genômica.^42^ Outros pesquisadores ecoam esse sentimento, destacando a necessidade crítica de políticas e práticas que promovam uma inclusão mais ampla dos participantes. Isso é essencial para maximizar o impacto global da pesquisa genética e da medicina de precisão.^43^ Um estudo recente de Patel et al.^44^ oferece notícias promissoras para abordar essa limitação fundamental. Os pesquisadores desenvolveram um novo escore genético para DAC integrando dados de associação de todo o genoma com informações de um conjunto diversificado de mais de 269.000 casos de DAC em várias ascendências. Esse escore superou significativamente todos os PRSs para DAC existentes em análises que incluíram indivíduos de várias etnias. Mais estudos são necessários para replicar esses achados.

Outra limitação dos PRSs é que características complexas são influenciadas por inúmeras variações genéticas comuns, cada uma com efeitos individuais mínimos. Essas variantes geralmente estão em LD com SNPs não causais próximos, dificultando a distinção entre elas. Para enfrentar esse desafio, Zheng et al.^45^ desenvolveram recentemente um modelo chamado SBayesRC. Esse modelo aplica anotações genômicas funcionais de SNPs candidatos, incorporando informações biológicas para diferenciar variantes causais prováveis daquelas irrelevantes. Notavelmente, o SBayesRC demonstrou uma melhora significativa na precisão da previsão. Conseguiu um aumento de 14% para 28 características e doenças complexas, e uma melhoria ainda maior, de 34%, na previsão média de ancestralidade para 18 características bem estudadas.^45^

Além disso, é importante reconhecer que vários fatores de risco que contribuem para o desenvolvimento de doenças cardiovasculares estão além do escopo dos escores poligênicos. Embora os PRSs possam estimar o risco de doença, eles podem não identificar com precisão os indivíduos que permanecerão saudáveis.^12^ A acurácia preditiva dos PRSs pode variar significativamente com base em fatores como sexo e idade.^46^

Um estudo recente sugere que a incorporação de determinantes sociais da saúde, fatores de estilo de vida e PRS em avaliações de risco pode melhorar significativamente a previsão de DAC em comparação ao uso isolado do PRS.^47^ Pesquisadores usando dados do UK Biobank verificaram que escores tanto de PRS quanto de determinantes sociais de saúde estavam correlacionadas com o risco clínico de DAC. Porém, uma medida combinada superou significativamente ambos os escores individuais. Ao analisar dados de mais de 471.000 indivíduos inicialmente saudáveis, o estudo mostrou que a combinação desses fatores levou a uma melhor previsão do risco de DAC, permitindo intervenções preventivas mais precoces.^47^ Apesar de sua contribuição potencial para a compreensão do desenvolvimento de DAC, os determinantes sociais da saúde e os PRSs ainda não estão incluídos nos modelos atuais de previsão de risco. Ademais, a extensão das disparidades entre os PRSs e outras abordagens importantes, como histórico médico familiar, ainda carece de clareza. Por fim, a aplicabilidade clínica atual dos modelos de PRS é limitada à identificação de populações de alto risco determinadas por percentis superiores de suscetibilidade genética.

Young et al.^48^ identificaram variações ambientais como outro fator que pode limitar a precisão dos PRSs. Essas variações ambientais podem interagir com genes, fazendo com que os tamanhos de efeito das variantes genéticas flutuem. Para abordar essa limitação, os pesquisadores enfatizam a importância de desconstruir os sinais de GWAS. Ao separar esses sinais, podemos identificar os componentes que levam a previsões mais generalizáveis, ou seja, previsões mais precisas em diferentes ambientes. Além disso, conforme afirmado pela American Heart Association,^49^ é crucial que a utilidade dos PRSs seja equilibrada e responsável, levando em consideração quaisquer riscos potenciais. Como a maioria dos estudos estima o prognóstico ou os efeitos do tratamento usando análises retrospectivas, a estimação dos danos potenciais ainda representa um desafio. Andreoli et al.^50^ realizaram uma revisão sistemática investigando as considerações éticas em torno do uso clínico dos PRSs. A análise dos autores identificou várias preocupações importantes que merecem atenção à medida que esses escores são integrados à prática médica. Uma preocupação central envolve o desenvolvimento de políticas e práticas clínicas que garantam acesso equitativo aos benefícios potenciais dos PRSs para todos os pacientes. Além disso, os autores destacam o risco potencial dos PRSs exacerbar as disparidades de saúde existentes.

## Considerações finais

Conforme discutido nas seções anteriores, o uso de PRSs é promissor tanto para prevenção primária quanto secundária (Figura Central), mas há diferenças entre elas, como será discutido abaixo. Um resumo dos estudos incluídos na presente revisão é apresentado na Tabela 1.

**Tabela 1:** – Características dos estudos incluídos e seus achados principais

Estudo	Ano	Desenho	Região	Participantes (N)	Achados principais
Wünnemann et al.^15^	2019	Coorte	América do Norte	11.021	Dois PRS foram eficazes na identificação de indivíduos franco-canadenses com alto risco de desenvolver DAC.
Tikkanen et al.^23^	2013	Coorte	Europa	24.124	A incorporação de informações genéticas na avaliação de risco melhorou a prevenção e o manejo de DAC
Khera et al.^24^	2018	Coorte	Europa	288.978	Indivíduos no 1% mais alto da distribuição do PRS para mais de 6 milhões de variantes tiveram quase 5 vezes mais probabilidade de desenvolver DAC.
Inouye et al.^25^	2018	Coorte	Europa	482.629	A previsão de risco genômico pode identificar efetivamente indivíduos com alto risco de desenvolver DAC.
Lu et al.^26^	2022	Coorte	China	41.271	O uso de um PRS melhorou significativamente a identificação de indivíduos com alto risco de desenvolver DAC na população chinesa.
Mega et al.^27^	2015	Coorte	Europa	48.421	Indivíduos com maior predisposição genética a eventos coronários apresentaram maior redução no risco quando tratados com estatinas.
Tada et al.^28^	2021	Coorte	Europa	23.595	PRS foi capaz de avaliar com precisão o risco de DAC sem considerar o histórico familiar, ressaltando sua utilidade como uma ferramenta independente de previsão de risco.
Marston et al.^29^	2023	Coorte	Europa	330.201	O uso de um PRS para DAC previu efetivamente o risco em estratégias de prevenção primária.
Natarajan et al.^30^	2017	Coorte	Europa	4.392	O uso de PRS identificou um subgrupo de indivíduos com maior carga de aterosclerose que podem se beneficiar mais da terapia com estatinas para prevenção primária.
Oni-Orisan et al.^31^	2022	Coorte	América do Norte	32.196	Indivíduos com PRS mais alto apresentaram uma redução mais significativa no risco de infarto do miocárdio com o uso de estatina em comparação com aqueles com PRS mais baixo.
Cornelissen et al.^32^	2024	Coorte	América do Norte	954	Indivíduos com PRS mais alto tenderam a desenvolver placas ateroscleróticas mais extensas e complexas, caracterizadas por características mais desfavoráveis, incluindo uma maior propensão à ruptura.
Cho et al.^33^	2023	Coorte	Europa	7.024	Fatores genéticos interagiram com estilo de vida e fatores clínicos, influenciando a probabilidade de recorrência de eventos coronários.
Qin et al.^35^	2024	Coorte	China	1.776	Indivíduos asiáticos com PRS mais alto apresentaram um risco mais alto de mortalidade por todas as causas.
Howe et al.^36^	2020	Coorte	Europa	403.395	O PRS ajudou a identificar pacientes com DAC estabelecida que tinham maior probabilidade de apresentar eventos adicionais.
Thompson et al.^37^	2022	Coorte	Oceania	4.932	Variantes genéticas não previram significativamente a ocorrência de DAC após o diagnóstico da doença.
Kiflen et al.^38^	2022	Custo-efetividade	Europa	96.116	O uso de PRS para orientar a terapia com estatina foi custo-efetivo em comparação com estratégias baseadas apenas em fatores de risco tradicionais.
Mujwara et al.^39^	2023	Custo-efetividade	América do Norte	47.108	A implementação de um PRS em um programa de prevenção no local de trabalho provou ser custo-efetiva.
Mujwara et al.^40^	2022	Custo-efetividade	América do Norte	47.108	A integração de um PRS como um fator de risco adicional em equações de coorte agrupadas aumentou a acurácia da estratificação de risco de DAC, mantendo custo-efetividade.

DAC: doença arterial coronariana; PRS: escore de risco poligênico.

Na prevenção primária da DAC, a aplicação de PRSs é uma estratégia racional, visando reduzir o risco do primeiro evento, o que, em teoria, caracterizaria o indivíduo como de menor risco. Nesse contexto, medidas preventivas personalizadas podem ser altamente eficazes, incluindo mudanças no estilo de vida e monitoramento médico mais rigoroso. Por fim, o impacto potencial dos PRSs na prevenção primária é significativo, pois permite intervenções antes do desenvolvimento da doença.

Na prevenção secundária, os PRSs ajudam a estratificar o risco em pacientes com DAC estabelecida, otimizando o tratamento e identificando aqueles com maior risco de eventos futuros. Isso permite medidas preventivas mais intensivas, como ajustes de tratamento ou acompanhamento frequente.

Os PRSs são promissores para revolucionar o atendimento aos pacientes por aprimorar a estratificação de risco e melhorar os desfechos clínicos. No entanto, sua aplicação requer uma abordagem multifacetada. Primeiramente, considerações éticas e psicossociais exigem uma avaliação cuidadosa. Em segundo lugar, os PRSs devem ser vistos como complementares aos fatores de risco estabelecidos e aos dados clínicos. A integração com essas ferramentas existentes facilitará, em última análise, o desenvolvimento de estratégias personalizadas de manejo de pacientes. Finalmente, a validação rigorosa por meio de estudos prospectivos é necessária para confirmar a verdadeira eficácia dos PRSs.

## Conclusões

Avanços consideráveis têm sido feitos na área da genômica nas últimas décadas. Os PRSs representam a combinação de vários fatores de risco causais, em oposição a um único caminho que leva à doença. Indivíduos com risco genético aumentado para DAC, seja poligênica ou monogênica, podem se beneficiar de tratamentos e abordagens abrangentes de redução de risco. A aplicação dos PRSs traz a possibilidade de quantificar o risco de um indivíduo desenvolver DAC, permitindo a prevenção precoce e/ou o início de um tratamento específico. Ao contrário dos altos custos inerentes a grandes ensaios clínicos randomizados, o uso dos PRSs tem o potencial de permitir um enriquecimento preditivo ou prognóstico substancial e pode ter um impacto profundo ao inaugurar uma nova era no desenvolvimento clínico. Finalmente, os PRSs mostram-se promissores na prevenção de DAC, abrangendo tanto a prevenção primária quanto a secundária. No entanto, é importante reconhecer as distinções entre essas duas aplicações para maximizar a efetividade dessa ferramenta.
