# Supplementary material for: Escores de Risco Poligênico: O Próximo Passo para Melhorar a Estratificação de Risco na Doença Arterial Coronariana?
Source: Arq Bras Cardiol. 2024 Sep 12;121(9):e20240252. [Article in Portuguese] doi: 10.36660/abc.20240252 (PMC11495647; doi:10.36660/abc.20240252)
Supplement: Translation

# Translation: Polygenic Risk Scores: The Next Step for Improved Risk Stratification in Coronary Artery Disease?

**DOI:** 10.36660/abc.20240252i

**Authors:** Ricardo Stein, Filipe Ferrari, Diego García-Giustiniani

**Affiliations:** 1 Brazil Programa de Pós-Graduação em Cardiologia e Ciências Cardiovasculares, Universidade Federal do Rio Grande do Sul, Porto Alegre, RS – Brazil; 2 Brazil Departamento de Medicina Interna, Universidade Federal do Rio Grande do Sul, Porto Alegre, RS – Brazil; 3 Spain Instituto de Investigación Biomédica de A Coruña (INIBIC), Coruña, Galicia – Spain

**Keywords:** Coronary Artery Disease, Risk, Human Genome, Cardiovascular Diseases

## Abstract

Despite significant advances in the management of coronary artery disease (CAD) and reductions in annual mortality rates in recent decades, this disease remains the leading cause of death worldwide. Consequently, there is an ongoing need for efforts to address this situation. Current clinical algorithms to identify at-risk patients are particularly inaccurate in moderate-risk individuals. For this reason, the need for ancillary tests has been suggested, including predictive genetic screening. As genetic studies rapidly expand and genomic data becomes more accessible, numerous genetic risk scores have been proposed to identify and evaluate an individual’s susceptibility to developing diseases, including CAD. The field of genetics has indeed made substantial contributions to risk prediction, particularly in cases where children have parents with premature CAD, resulting in an increased risk of up to 75%. The polygenic risk scores (PRSs) have emerged as a potentially valuable tool for understanding and stratifying an individual’s genetic risk. The PRS is calculated as a weighted sum of single-nucleotide variants present throughout the human genome, identifiable through genome-wide association studies, and associated with various cardiometabolic diseases. The use of PRSs holds promise, as it enables the development of personalized strategies for preventing or diagnosing specific pathologies early. Furthermore, it can complement existing clinical scores, increasing the accuracy of individual risk prediction. Consequently, the application of PRSs has the potential to impact the costs and adverse outcomes associated with CAD positively. This narrative review provides an overview of the role of PRSs in the context of CAD.

## Introduction

For decades, various clinical algorithms have been developed to identify patients at risk for coronary artery disease (CAD) and to formulate primary prevention strategies. However, these algorithms prove less effective in individuals at intermediate risk.^1^ Therefore, in recent years, additional tests known as risk enhancers or risk modifiers have been investigated to more accurately identify this specific risk group. Some of these ancillary tests include apolipoprotein B, lipoprotein (a), or measures of inflammation, such as high-sensitivity C-reactive protein or coronary artery calcium score.^2-6^ These clinical enhancers must meet the condition of being independently associated with CAD and conferring at least a two-fold increased risk of disease.^1,7^ In addition to the investigated clinical enhancers, earlier results from the Framingham Heart Study offspring cohort demonstrated that a history of premature CAD was associated with a two-fold increase in the likelihood of cardiovascular disease after adjustment for traditional clinical risk factors. This suggests a clear heritable basis for cardiovascular disease.^8^

Recent advances in the identification of genomic data, particularly in the last decades, have presented significant opportunities for the development of independent genetic predictors. The elucidation of the genetic basis of diseases, coupled with recent studies revealing a nearly two-fold increase in risk levels among populations with high percentile risk scores, holds promise for integrating these scores into clinical practice. This integration can be achieved by incorporating polygenic risk scores (PRSs) into the existing risk assessment models.^1^

This narrative review explores the current understanding of PRSs in relation to CAD, and we aim to highlight both the potential benefits and limitations of PRSs for CAD risk assessment, while also considering their cost-effectiveness.

## Methods

We employed a narrative approach, allowing for a qualitative synthesis of relevant studies. Extracted information was then organized to construct a cohesive narrative on the role of PRSs in the context of CAD diagnosis and management. A PubMed/MEDLINE literature search was conducted for studies published from inception to March 26, 2024. The following search strategy was utilized: (polygenic risk scores OR genetic risk scores) and (coronary artery disease OR CAD). We hand-searched the reference lists of all included studies to identify other potential articles. Of the 1,017 articles retrieved, 19 were included, comprising 15 cohort studies, 3 cost-effectiveness analyses, and 1 guideline.

### Polygenic risk scores – definition and concept

The PRS is a computed sum of risk alleles carried by an individual, serving as a predictive tool for assessing risk.^9^ In short, PRSs are measures that quantify an individual’s genetic predisposition to a disease by considering multiple genetic variants across the entire genome. These scores integrate information from a large number of genetic markers, with each contributing a small portion to the overall risk. Their construction involves the use and validation of single-nucleotide variants (SNVs), representing alterations of a single nucleotide at a specific position in the genome, primarily obtained from genome-wide association studies (GWAS).

In simpler terms, a PRS is a computational algorithm that incorporates information derived from SNVs. Initially, risk scores were based on unweighted calculations, but this approach has been replaced by weighted scores, recognizing the varying effects of individual SNVs on specific diseases.^10^ PRSs alone do not account for critical factors involved in developing a particular disease, such as environmental influences. Therefore, it should be considered as one of several contributing independent risks, and it cannot definitively predict whether an individual will develop the disease.^11^

To provide context, it is essential to understand that, even if an individual possesses a strong genetic predisposition to drug addiction, it becomes irrelevant if he or she never initiates drug use.^12^

### Construction of Polygenic Risk Scores

In essence, a PRS is constructed based on GWAS using genotyped single-nucleotide polymorphisms (SNPs). Each SNP has attributes including identifier, position, risk classification, effect size, confidence measure, and p-value. PRS calculation involves summing risk alleles (0, 1, or 2) from target data, weighted by the effect sizes of the risk alleles. Effect sizes are represented as log (odds ratio) for binary traits or as regression slopes for continuous traits.^13^ After identifying SNPs and their weights, PRS is computed by summing their contributions. A higher PRS indicates a higher genetic predisposition, while lower scores suggest a lower risk.

Weighted PRSs are preferred for better predictive accuracy over unweighted PRSs.^14^The chosen p-value threshold determines the number of SNPs in a PRS model, impacting sensitivity and specificity in prediction. Hence, it is common to test different PRS sizes with various p-value thresholds during development.

Another crucial consideration is to assess whether the variants in the model are in linkage disequilibrium (LD), indicating co-inheritance. LD adjustment helps prevent the overrepresentation of genetic variants in high LD regions, essential for PRS performance. Modern computational tools like LDPred and meta-genetic risk score (metaGRS) address it.^15,16^ For instance, metaGRS offers better risk discrimination compared to older calculators based on selected SNPs. It captures a larger proportion of CAD heritability, around 27%, with significant improvements in risk prediction.^15^

The next step involves finding the optimal PRS model by testing various p-value thresholds, and evaluating different models through association studies. The top-performing score is then validated using standard epidemiological measures such as odds ratios or hazards ratios per standard deviation (SD) change in PRS (for binary diseases), the proportion of phenotypic variation explained (R2 or pseudo-R2), the area under the receiver operating characteristic curve or C statistic, and the p-value of association.^17^ This approach enables the classification of individuals into low, intermediate, or high-risk categories. Individuals with fewer disease-related polymorphisms typically fall into the low-risk category. It is important to note that PRSs can vary across different diseases and populations. Figure 1 summarizes the essential aspects of a PRS analysis.

**Figure 1:** – Essential aspects of a polygenic risk score analysis. AUC: area under the receiver operating characteristic curve; GWAS: genome-wide association studies; LD: linkage disequilibrium; OR: odds ratio; PRS: polygenic risk score; SNPs: single nucleotide polymorphisms.

### Genetic predisposition to coronary artery disease

Genetic factors significantly contribute to the development of CAD, with evidence dating back to the 1950s, when evidence highlighted the importance of hereditary factors in this regard.^18^ In this context, around one third of CAD patients exhibit a positive family history, correlating with approximately a 1.5-fold heightened risk of CAD over their lifetimes.^19,20^A large-scale study from the Framingham Heart Study involving over 2,300 men and women with an average age of 44 years showed a significantly increased risk of cardiovascular events for individuals with a parent who had early-onset cardiovascular disease (father before 55 years, mother before 65 years).^8^ This finding aligns with the concept that a family history of early-onset cardiovascular disease (before age 50) is associated with a higher risk of death from CAD. A recent study by Taylor et al. further supports this, with over 6,200 participants followed for an average of 15 years.^21^ In their study, over 40% had at least one parent with a history of cardiovascular disease, while slightly more than half had no known family history. The results demonstrated that a family history of cardiovascular disease increased the risk of developing future cardiovascular disease by 1.7 times (hazard ratio [HR]: 1.71; 95% confidence interval [CI], 1.33 to 2.21; p < 0.001).

Furthermore, the relationship between parental history of CAD and the risk of developing the disease was collected by the observed association between CAD and other common risk factors, such as high blood pressure, high cholesterol levels, and smoking. Evidence was also found to suggest a correlation between family history of CAD and markers of subclinical atherosclerosis (e.g., coronary artery calcium, carotid intima thickness, and vascular function), even after consideration of traditional risk factors.^22^ This implies that these individuals can be identified as strong candidates for evaluation of subclinical cardiovascular disease, thereby assisting in improving treatment goals and risk management.

These data suggest that family history of CAD is significant as a risk factor that should be evaluated in conjunction with other recognized risk factors when determining the likelihood of developing CAD.

### Polygenic Risk Scores as a complementary tool in coronary artery disease primary prevention

#### Identification of individuals at risk for coronary artery disease

The identification of individuals undergoing primary prevention who are at high risk for CAD is crucial, as it allows for better screening or preventive therapies. Recognizing cardiovascular risk represents the first step in determining the approach to individual treatment for primary prevention. Within this context, numerous tools have been devised to aid in this evaluation, including PRSs.

Tikkanen et al.^23^ developed a genetic risk score and evaluated its correlation with incident cardiovascular disease events over 12 years. Analyzing 28 genetic variants associated with CAD in a sample size of over 24,000 individuals from four population-based prospective cohorts, they compared the genetic risk score with conventional risk factors and family history. The study revealed that the genetic risk score significantly enhanced the ability to predict CAD risk for new events. Individuals within the top 10% of the genetic risk score, based on 28 SNPs, exhibited a two-fold increase in CAD risk compared to those in the middle 20%.

In the study by Khera et al.,^24^ a genome-wide set of PRS was calculated using the LDPred algorithm for various diseases, including CAD. The top 1% of the distribution of the 6.6 million PRS variants had almost five times greater chances of developing CAD (odds ratio: 4.83; 95% CI, 4.25 to 5.46). The odds ratio was calculated by comparing those with elevated genome-wide polygenic scores (a quantitative predictor of inherited risk) to the rest of the population in a logistic regression model adjusted for age, sex, genotyping matrix, and the first four principal components of ancestry. Inouye et al.^25^ conducted a meta-analysis of approximately 500,000 individuals to develop a genomic risk score for CAD based on over 1.7 million genetic variants. They discovered that these variants accounted for 26.8% of the heritability of this disease. Moreover, their findings unveiled that individuals classified in the highest PRS quintile (top 20%) exhibited a four-fold increased risk of CAD (HR: 4.17) compared with those in the bottom 20%. The study further showcased that the combined genomic risk score proved to be a more reliable predictor of CAD compared to various well-established risk factors, including high cholesterol and hypertension. Both the genome-wide polygenic score and metaGRS have been validated in the French-Canadian population.^15^ The authors confirmed that the PRS can identify approximately 6% to 7% of the population at equal or greater risk of CAD than carriers of a monogenic familial hypercholesterolemia mutation.^15^ These significant findings underscore the complementary predictive capabilities of the PRS alongside established risk factors for CAD, rather than advocating for their replacement.

Lu et al.^26^ developed a PRS with 540 genetic variants for CAD, evaluating its clinical utility in primary prevention in a training set with 2,800 CAD cases and 2,055 controls. During an average follow-up of 13 years, 1,303 incident cases of CAD were identified. Those with elevated PRS (top 20%) had about a three-fold increased risk of CAD compared with the lowest 20% (HR: 2.91; 95% CI, 2.43 to 3.49). Adding the PRS to the clinical risk score resulted in a modest but significant improvement in C statistic (1%) and a net improvement in reclassification (3.5%).^26^ This study highlighted the considerable potential of identifying high-risk individuals for targeted interventions in clinical practice.

Extremely elevated PRS for CAD may also play an important role in early statin therapy. Mega et al.^27^ investigated the association between a risk score based on 27 genetic variants and the occurrence of new or recurrent CAD events while considering clinical predictors. The researchers gathered data from a community-based cohort and four large randomized clinical trials (JUPITER, ASCOT, CARE, and PROVE IT-TIME 22), involving over 48,000 individuals and nearly 3,500 events.^27^ Among individuals in the primary prevention populations, higher risks of CAD incidence were observed in the intermediate and high genetic risk categories, with hazard ratios of 1.31 and 1.72, respectively, compared to the low genetic risk category (p < 0.0001 for both). Interestingly, the study also found that statin therapy significantly reduced the relative risk of events in the high genetic risk category compared to the low risk category (48% vs. 13%, respectively). Additionally, a three-fold gradient in the number needed to treat (NNT) was observed in primary prevention studies between individuals in the highest genetic risk categories and those in the lowest genetic risk category. Specifically, among participants in the JUPITER trial enrolled in primary prevention, the NNT to prevent an ischemic cardiovascular event over 10 years was 66, 42, and 25 for individuals in the low, intermediate, and high genetic risk score groups, respectively. These findings highlight that individuals with a higher genetic risk for CAD derive the most significant clinical benefits from statin use. Furthermore, the study conducted by Tada et al.^28^ indicates a potentially greater advantage in utilizing PRS for young individuals. In this research, the incorporation of 23 SNPs into a PRS enhanced the prediction of ischemic heart disease risk with a median follow-up of 14.4 years, irrespective of the family history of self-reported cardiovascular disease. The study involved 23,595 participants from the Malmö Diet and Cancer Study, a prospective, population-based study.

Another study based on findings regarding statins and CAD demonstrated that a PRS for CAD had a stronger predictive ability in younger individuals.^29^ The CAD PRS consisted of 241 significant genetic variations distributed across the genome. To estimate the 10-year risk of atherosclerotic cardiovascular disease, the researchers employed pooled cohort equations, which classified subjects into low (< 5%), borderline (5 to < 7.5%), intermediate (7.5 to < 20%), or high-risk (≥ 20%) categories. The analysis revealed a strong association between the CAD PRS and the risk of myocardial infarction across all age groups. However, the predictive power was notably more robust in younger individuals (age < 50 years: HR per 1 SD of PRS, 1.72; 95% CI, 1.56 to 1.89; age 50 to 60 years: HR, 1.46; 95% CI, 1.38 to 1.53; age > 60 years: HR, 1.42; 95% CI, 1.37 to 1.48; p for interaction < 0.001). In patients under 50 years old, those with a high PRS exhibited a three to four times higher risk of myocardial infarction compared to those in the low PRS category.^29^ Consistent with these findings, a study that included participants from the randomized controlled trial of primary prevention with statin therapy (WOSCOPS; N = 4,910) and two observational cohort studies (CARDIA and BioImage; N = 1,154 and N = 4,392, respectively)^30^ reported that individuals at high genetic risk had a greater burden of subclinical atherosclerosis and derived greater relative and absolute benefits from statin therapy to prevent a first CAD event. Furthermore, the American Guideline for Primary Prevention of CAD recommends preventive statin therapy for individuals carrying a rare monogenic familial hypercholesterolemia mutation.^7^

Results from a study involving a real-world cohort in primary prevention support rationalizing the use of CAD PRS as a precision medicine tool to further optimize the risk-benefit balance of statin therapy in combination with traditional risk factors for cardiovascular disease. The magnitude of statin efficacy became progressively more robust in groups with low (quintile 1; HR: 0.67; 95% CI, 0.47 to 0.97), intermediate (quintiles 2 to 4; HR: 0.56; 95% CI, 0.47 to 0.66), and high PRS (quintile 5; HR: 0.41; 95% CI, 0.31 to 0.53), with a smaller benefit of statins in the group with low PRS (p = 0.01 comparing high versus low).^31^

Finally, an autopsy study investigated the connections between the PRS and the severity of atherosclerosis at a histopathological level in individuals who suffered sudden death. By analyzing a sample consisting of over 900 cases, with a mean age of 48 years, the authors discovered that those in the highest PRS quintile had more severe atherosclerosis compared to those in the lowest quintile, as well as a higher incidence of calcification and thin-cap fibroatheroma, all with statistical significance.^32^ Even after adjusting for traditional CAD risk factors, those in the highest PRS quintile had higher odds of having severe atherosclerosis (defined as ≥ 75% stenosis; adjusted odds ratio: 3.8; 95% CI, 2.1 to 6.8; p < 0.001) and plaque rupture (adjusted odds ratio: 4.1; 95% CI, 2.3 to 7.2; p < 0.001). Furthermore, individuals in the highest quintile were more likely to have CAD-associated causes of death, especially among younger people (age ≤ 50 years; adjusted odds ratio: 4.1; 95% CI, 2.0 to 8.3; p < 0.001).^32^ These results provide solid evidence of an association between PRS and advanced atherosclerosis, suggesting that PRS could serve as a valuable tool for stratifying CAD risk, especially in younger populations.

## Polygenic Risk Scores in secondary prevention for coronary artery disease

Secondary prevention of coronary heart disease aims to prevent the recurrence of coronary events. A high degree of adherence to secondary prevention measures, especially intensive lifestyle changes and medication use, can result in a significant reduction in the incidence of recurrent coronary events. The PRS, in turn, can also provide a valuable strategy to aid in identifying the risk of a new cardiovascular event.

A cohort from the UK Biobank, comprising over 7,000 middle-aged adults (mean age: 62 years) diagnosed with established CAD, was monitored for a median duration of approximately 12 years. This investigation identified CAD PRS (C index: 0.58; 95% CI, 0.57 to 0.59) as one of the most robust predictors of CAD recurrence. Both history of an initial premature CAD event and an elevated CAD PRS were significant and mutually reinforcing risk factors for recurrent CAD. Importantly, CAD PRS exhibited an independent association with a 12% heightened risk of recurrent CAD events (HR: 1.12; 95% CI, 1.05 to 1.19).^33^ As highlighted in an editorial, it is important to underscore certain limitations inherent in this study. Firstly, the models employed lacked an evaluation of left ventricular function, the presence of heart failure, concurrent valve disease, the anatomical extent of CAD, and associated atherosclerotic disease burden in other vascular beds. The incorporation of these variables could significantly enhance risk prediction. Moreover, although the inclusion of the PRS contributed to an overall modest C index of 0.676, the discriminatory capacity exhibited only limited and incremental improvement compared to traditional risk factors alone (C index of 0.644).^34^ Even with the described limitations, these findings emphasize the potential of the genetic risk score in both predicting and preventing future CAD.

In a cohort of 1,776 Chinese patients with CAD who were followed for up to 11 years, genetic susceptibility to CAD and its traditional risk factors (e.g., heart failure, angina, diabetes, and LDL cholesterol) were evaluated to predict death from all causes. The results showed that the integration of metaPRS for CAD and its risk factors was significantly associated with mortality. In this study, participants were divided into three groups based on quartiles of metaPRS scores. Patients with CAD in the third quartile exhibited a significantly higher cumulative incidence of all-cause mortality compared to those in the first quartile (HR: 3.99; 95% CI, 2.4 to 6.6) per SD increase (p = 9.10 × 10^8^). Additionally, patients with CAD and intermediate metaPRS scores also showed a significantly higher cumulative incidence of all-cause mortality compared to those in the first quartile (HR: 2.18; 95% CI, 1.3 to 3.6) per SD increase (p = 2.10 × 10^3^).^35^ In turn, Howe et al.^36^ conducted a study to investigate whether the PRS could stratify the risk of subsequent events in survivors of an ischemic cardiac event. They analyzed two subsamples from the UK Biobank: individuals with prevalent CAD cases (N = 10,287) and individuals without CAD (N = 393,108) as the baseline. The study revealed a significant difference in the associations between CAD PRS and cardiovascular events for individuals with and without previous CAD. In the group without CAD, there was strong evidence of a positive association between CAD PRS and a 33% increase in the risk for myocardial infarction, whereas this increase was 15% in the group with CAD. Consequently, the utility of PRS appears to be diminished in individuals undergoing secondary prevention. Other authors, including Thompson et al.^37^ have also found limited success in predicting future cardiovascular events (over a 10-year follow-up period) using genetic risk scores in patients with a history of acute coronary syndrome. These authors utilized specific SNPs associated with prevalent or incident ischemic heart disease in GWAS, as well as validated 27-SNP genetic risk scores based on these variants. The underlying reasons for these discrepancies remain unclear, and further research is required to determine the true contribution of genetic risk scores in predicting future risk among individuals who have already experienced a cardiovascular event.

## Cost-Effectiveness

As the application of PRSs may increase considerably across the world in the coming decades, an important topic to address is the cost-effectiveness of their utilization. Therefore, it is vital that the application of PRS, in addition to being effective, is cost-effective enough to allow for its use on a broad spectrum.

Kiflen et al.^38^investigated the cost-effectiveness of PRS concerning statin therapy, utilizing a UK Biobank cohort (N = 96,111; White and British descent) with an intermediate risk of cardiovascular disease, followed for at least 10 years. The base case analysis, with a genotyping cost of $70, resulted in an incremental cost-effectiveness ratio of $172,906 per quality-adjusted life year. In probabilistic sensitivity analysis, the intervention had an approximately 50% probability of being cost-effective at $179,100 per quality-adjusted life year. This study suggests that incorporating PRS alongside existing guidelines may be cost-effective for cardiovascular disease. Greater predictability combined with a decreased cost of PRS could further improve cost-effectiveness, providing an economic basis for its inclusion in clinical care.

Another study involving individuals aged 40 to 75 in the United States revealed that the incorporation of a CAD-PRS into a workplace cardiovascular disease prevention program proved cost-effective.^39^ The analysis showed that the inclusion of CAD-PRS incurred additional costs of over $53 per employee examined compared to the workplace cardiovascular prevention program without CAD-PRS, and over $575 compared to the absence of a workplace health program. The authors consequently assert that integrating polygenic testing into a workplace cardiovascular prevention program not only enhances the quality of life for employees, but also concurrently diminishes healthcare costs and mitigates financial losses in productivity for employers. The same researchers found that considering a genetic risk score (CAD-PRS) for heart disease led to better outcomes compared to using only a standard risk assessment tool. They saw a decrease in healthcare costs per person, improved quality of life, and fewer cases of CAD and stroke.^40^

## Limitations and areas of uncertainty

Despite representing a promising and rapidly expanding area, PRSs are not exempt from limitations, particularly concerning their external validity due to the predominant focus on individuals of European ancestry. Heterogeneity among different populations worldwide hampers the generalizability of PRSs and their usefulness across diverse ancestry groups, which might underrepresent certain populations, thus diminishing the utility of PRSs and limiting the applicability of the risk prediction tools.

One key challenge is the potential variation in the frequency and effects of genetic variants across different populations. This is supported by findings from previous research.^41^ Additionally, populations with a high degree of admixture, or mixing of ancestral origins, tend to have greater genetic diversity. This complexity can further hinder the accuracy of PRSs. To address these issues and improve the accuracy, it is essential to increase the diversity of participants in genomic research and to develop models that account for the specific genetic variations within each population group.

Consequently, conducting additional studies applying PRSs in non-European populations becomes imperative to confirm or refute their role as a predictor of future cardiovascular risk in these groups. A scientometric review by Mills and Rahal revealed a concerning trend: while ancestral diversity among participants in GWAS has increased over time, significant disparities remain.^42^ Even in 2017, a staggering 88% of participants were of European descent. Furthermore, a narrow geographic focus persists, with 72% of discoveries based on studies conducted in just three countries: the United States, the United Kingdom, and Iceland.^42^ These findings raise concerns about a potential “cycle of disadvantage” for underrepresented populations. Despite ongoing efforts, Mills and Rahal emphasize the ongoing challenge of increasing diversity in genomics research.^42^ Other researchers echo this sentiment, highlighting the critical need for policies and practices that promote broader participant inclusion. This is essential to maximize the global impact of genetic research and precision medicine.^43^A recent study by Patel et al.^44^ offers promising news in addressing this key limitation. The researchers developed a novel genetic score for CAD by integrating genome-wide association data with information from a diverse pool of over 269,000 CAD cases across various ancestries. This score significantly outperformed all existing CAD PRS in analyses that included individuals from multiple ethnicities. Further studies are warranted to replicate these findings.

Another limitation of PRSs is that complex traits are influenced by numerous common genetic variants, each with minimal individual effects. These variants are often in LD with nearby non-causal SNPs, making it difficult to distinguish between them. To address this challenge, Zheng et al.^45^ recently developed a model called SBayesRC. This model leverages functional genomic annotations of candidate SNPs, incorporating biological information to differentiate likely causal variants from irrelevant ones. Notably, SBayesRC demonstrated a significant improvement in prediction accuracy. It achieved a 14% increase for 28 complex traits and diseases, and an even greater improvement of 34% in average ancestry prediction for 18 well-studied traits.^45^

Moreover, it is important to acknowledge that numerous risk factors contributing to the development of cardiovascular disease lie beyond the scope of polygenic scores. While PRSs can estimate disease risk, they may not accurately identify individuals who will remain healthy.^12^ The predictive accuracy of the PRSs can vary significantly based on factors such as sex and age.^46^

A recent study suggests that incorporating social determinants of health, lifestyle factors, and PRS into risk assessments can significantly improve CAD prediction compared to using PRS alone.^47^ Researchers using UK Biobank data found that scores for both PRS and social determinants of health correlated with clinical CAD risk. However, a combined measure significantly outperformed both individual scores. By analyzing data from over 471,000 initially healthy individuals, the study showed that combining these factors led to better prediction of CAD risk, enabling earlier preventive interventions.^47^ Despite their potential contribution to understanding CAD development, social determinants of health and PRSs are still not included in current risk prediction models. Moreover, the extent of disparities between PRSs and other important approaches, such as family medical history, still lacks clarity. Lastly, the present clinical applicability of PRSs models is constrained to the identification of high-risk populations determined by upper percentiles of genetic susceptibility.

Young et al.^48^ identified environmental variations as another factor that can limit the accuracy of PRSs. These environmental variations can interact with genes, causing the effect sizes of genetic variants to fluctuate. To address this limitation, researchers emphasize the importance of decomposing the signals from GWAS. By separating these signals, we can identify the components that lead to more generalizable predictions, meaning predictions that are more accurate across different environments. Additionally, as stated by the American Heart Association,^49^ it is crucial that the utility of PRSs be balanced and responsible, taking into account any potential risks. Since the majority of studies estimate prognosis or treatment effects using retrospective analyses, estimating potential harm still poses a challenge. Andreoli et al.^50^ conducted a systematic review exploring the ethical considerations surrounding the clinical use of PRSs. Their analysis identified several key concerns that warrant attention as these scores become integrated into medical practice. A central concern involves developing policies and clinical practices that guarantee equitable access to the potential benefits of PRSs for all patients. Additionally, the authors highlight the potential risk of PRSs exacerbating existing health disparities.

## Final considerations

As discussed in previous sections, the use of PRSs is promising for both primary and secondary prevention (Central Illustration), but there are differences between them, as will be discussed below. A summary of the studies included in this review is presented in Table 1.

**Table 1:** – Characteristics of included studies with major findings

Study	Year	Design	Region	Participants (N)	Major findings
Wünnemann et al.^15^	2019	Cohort	North America	11,021	Two PRS were effective in identifying French-Canadian individuals at high risk of developing CAD.
Tikkanen et al.^23^	2013	Cohort	Europe	24,124	Incorporating genetic information into risk assessment has enhanced the prevention and management of CAD.
Khera et al.^24^	2018	Cohort	Europe	288,978	Individuals in the top 1% of the PRS distribution for over 6 million variants were nearly five times more likely to develop CAD.
Inouye et al.^25^	2018	Cohort	Europe	482,629	Genomic risk prediction can effectively identify individuals at a high risk of developing CAD.
Lu et al.^26^	2022	Cohort	China	41,271	The use of a PRS significantly enhanced the identification of individuals at high risk of developing CAD in the Chinese population.
Mega et al.^27^	2015	Cohort	Europe	48,421	Individuals with a higher genetic predisposition to coronary events experienced a greater reduction in risk when treated with statins.
Tada et al.^28^	2021	Cohort	Europe	23,595	PRS can accurately assess CAD risk without considering family history, underscoring its utility as an independent risk prediction tool.
Marston et al.^29^	2023	Cohort	Europe	330,201	The use of a PRS for CAD effectively predicted risk in primary prevention strategies.
Natarajan et al.^30^	2017	Cohort	Europe	4,392	The use of PRS identified a subgroup of individuals with a higher burden of atherosclerosis who may benefit most from statin therapy for primary prevention.
Oni-Orisan et al.^31^	2022	Cohort	North America	32,196	Individuals with a higher PRS experienced a more significant reduction in myocardial infarction risk with statin use compared to those with a lower PRS.
Cornelissen et al.^32^	2024	Cohort	North America	954	Individuals with a higher PRS tended to develop more extensive and complex atherosclerotic plaques, characterized by more unfavorable traits, including a greater propensity to rupture.
Cho et al.^33^	2023	Cohort	Europe	7,024	Genetic factors interacted with lifestyle and clinical factors, influencing the likelihood of recurrence of coronary events.
Qin et al.^35^	2024	Cohort	China	1,776	Asian individuals with a higher PRS faced an increased risk of all-cause mortality.
Howe et al.^36^	2020	Cohort	Europe	403,395	The PRS helped identify patients with established CAD who were most likely to experience additional events.
Thompson et al.^37^	2022	Cohort	Oceania	4,932	Genetic variants did not significantly predict the occurrence of CAD after the disease was diagnosed.
Kiflen et al.^38^	2022	Cost-effectiveness	Europe	96,116	Using PRS to guide statin therapy was cost-effective compared to strategies based solely on traditional risk factors.
Mujwara et al.^39^	2023	Cost-effectiveness	North America	47,108	Implementing PRS in a workplace prevention program proved to be cost-effective.
Mujwara et al.^40^	2022	Cost-effectiveness	North America	47,108	Integrating PRS as an additional risk factor into pooled cohort equations enhanced the accuracy of CAD risk stratification while remaining cost-effective.

CAD: coronary artery disease; PRS: polygenic risk score

In the primary prevention of CAD, the application of PRSs is a rational strategy, aiming to reduce the risk of the first event, which, in theory, would characterize the individual as being at lower risk. In this context, personalized preventive measures can be highly effective, including lifestyle changes and more rigorous medical monitoring. Lastly, the potential impact of PRSs on primary prevention is significant, as it allows interventions before disease development.

In secondary prevention, PRSs help to stratify risk in patients with established CAD, optimizing treatment and identifying those at the highest risk of future events. This allows for more intensive preventative measures, such as treatment adjustments or frequent follow-up.

PRSs hold promise for revolutionizing patient care by enhancing risk stratification and improving clinical outcomes. However, their application requires a multifaceted approach. Firstly, ethical and psychosocial considerations necessitate careful evaluation. Secondly, PRSs should be viewed as complementary to established risk factors and clinical data. Integration with these existing tools will ultimately facilitate the development of personalized patient management strategies. Finally, rigorous validation through prospective studies is necessary to confirm the true effectiveness of PRSs.

## Conclusions

Considerable advances have been made in the area of genomics in recent decades. PRSs represent the combination of several causal risk factors, as opposed to a single pathway leading to the disease. Individuals with an increased genetic risk for CAD, whether polygenic or monogenic, may benefit from comprehensive risk reduction approaches and treatment. The application of PRSs brings the possibility of quantifying an individual’s risk for developing CAD, allowing for early prevention and/or initiation of specific treatment. Unlike the high costs inherent in large randomized clinical trials, PRSs have the potential to enable substantial predictive or prognostic enrichment and could have a profound impact by opening a new era in clinical development. Finally, PRSs show promise in preventing CAD, encompassing both primary and secondary prevention. However, it is important to discern the distinctions between these two applications to maximize the effectiveness of this tool.
